# Castanet: a pipeline for rapid analysis of targeted multi-pathogen genomic data

**DOI:** 10.1093/bioinformatics/btae591

**Published:** 2024-10-03

**Authors:** Richard Mayne, Shannah Secret, Cyndi Geoghegan, Amy Trebes, Kai Kean, Kaitlin Reid, Gu-Lung Lin, M Azim Ansari, Mariateresa de Cesare, David Bonsall, Ivo Elliott, Paolo Piazza, Anthony Brown, James Bray, Julian C Knight, Heli Harvala, Judith Breuer, Peter Simmonds, Rory J Bowden, Tanya Golubchik

**Affiliations:** Nuffield Department of Medicine, Peter Medawar Building for Pathogen Research, University of Oxford, Oxfordshire OX1 3SY, United Kingdom; Radcliffe Department of Medicine, University of Oxford, West Wing John Radcliffe Hospital, Oxfordshire OX3 9DU, United Kingdom; Microbiology Services, NHS Blood and Transplant, London NW9 5BG, United Kingdom; Centre for Human Genetics, University of Oxford, Oxfordshire OX3 7BN, United Kingdom; Genewiz UK Ltd, Azenta Life Sciences, Oxfordshire OX14 1SG, United Kingdom; Oxford Genomics Centre, University of Oxford, Oxfordshire OX3 7BN, United Kingdom; Nuffield Department of Medicine, Peter Medawar Building for Pathogen Research, University of Oxford, Oxfordshire OX1 3SY, United Kingdom; Nuffield Department of Medicine, Peter Medawar Building for Pathogen Research, University of Oxford, Oxfordshire OX1 3SY, United Kingdom; Oxford Vaccine Group, University of Oxford, Oxfordshire OX3 7LE, United Kingdom; Nuffield Department of Medicine, Peter Medawar Building for Pathogen Research, University of Oxford, Oxfordshire OX1 3SY, United Kingdom; National Facility for Genomics, Human Technopole, Viale Rita Levi-Montalcini, Milan 20157, Italy; Nuffield Department of Medicine, Peter Medawar Building for Pathogen Research, University of Oxford, Oxfordshire OX1 3SY, United Kingdom; Centre for Tropical Medicine and Global Health, University of Oxford, Oxfordshire OX3 7LE, United Kingdom; Centre for Human Genetics, University of Oxford, Oxfordshire OX3 7BN, United Kingdom; Nuffield Department of Medicine, Peter Medawar Building for Pathogen Research, University of Oxford, Oxfordshire OX1 3SY, United Kingdom; Department of Biology, University of Oxford, Oxfordshire OX1 3SY, United Kingdom; Oxford Genomics Centre, University of Oxford, Oxfordshire OX3 7BN, United Kingdom; Chinese Academy of Medical Science Oxford Institute, University of Oxford, Oxfordshire OX3 7BN, United Kingdom; NIHR Oxford Biomedical Research Centre, University of Oxford, John Radcliffe Hospital, Oxfordshire OX3 9DU, United Kingdom; Radcliffe Department of Medicine, University of Oxford, West Wing John Radcliffe Hospital, Oxfordshire OX3 9DU, United Kingdom; Microbiology Services, NHS Blood and Transplant, London NW9 5BG, United Kingdom; Institute of Child Health, University College London, London WC1N 1EH, United Kingdom; Nuffield Department of Medicine, Peter Medawar Building for Pathogen Research, University of Oxford, Oxfordshire OX1 3SY, United Kingdom; Genomics Lab, The Walter and Eliza Hall Institute of Medical Research, Victoria 3052, Melbourne, Australia; Nuffield Department of Medicine, Peter Medawar Building for Pathogen Research, University of Oxford, Oxfordshire OX1 3SY, United Kingdom; Sydney Infectious Diseases Institute, Faculty of Medicine and Health, University of Sydney, New South Wales 2050, Sydney, Australia

## Abstract

**Motivation:**

Target enrichment strategies generate genomic data from multiple pathogens in a single process, greatly improving sensitivity over metagenomic sequencing and enabling cost-effective, high-throughput surveillance and clinical applications. However, uptake by research and clinical laboratories is constrained by an absence of computational tools that are specifically designed for the analysis of multi-pathogen enrichment sequence data. Here we present an analysis pipeline, Castanet, for use with multi-pathogen enrichment sequencing data. Castanet is designed to work with short-read data produced by existing targeted enrichment strategies, but can be readily deployed on any BAM file generated by another methodology. Also included are an optional graphical interface and installer script.

**Results:**

In addition to genome reconstruction, Castanet reports method-specific metrics that enable quantification of capture efficiency, estimation of pathogen load, differentiation of low-level positives from contamination, and assessment of sequencing quality. Castanet can be used as a traditional end-to-end pipeline for consensus generation, but its strength lies in the ability to process a flexible, pre-defined set of pathogens of interest directly from multi-pathogen enrichment experiments. In our tests, Castanet consensus sequences were accurate reconstructions of reference sequences, including in instances where multiple strains of the same pathogen were present. Castanet performs effectively on standard computers and can process the entire output of a 96-sample enrichment sequencing run (50M reads) using a single batch process command, in $<$2 h.

**Availability and implementation:**

Source code freely available under GPL-3 license at https://github.com/MultipathogenGenomics/castanet, implemented in Python 3.10 and supported in Ubuntu Linux 22.04. The data underlying this article are available in Europe Nucleotide Archives, at https://www.ebi.ac.uk/ena/browser/view/PRJEB77004.

## 1 Introduction

The popularity of metagenomics for sequencing multiple organisms from clinical samples has generated increased interest in strategies to enrich metagenomic libraries for organisms of interest, both to reduce per-sample cost and to improve sensitivity. Targeted enrichment by hybrid capture, also called ‘targeted sequencing’ or ‘bait capture’ is an increasingly popular method that involves the hybridization of the metagenomic library with a potentially large panel of oligonucleotide probes, followed by amplification of the probe-bound sequences to 100–1000× above the metagenomic background. Targeted enrichment has been shown to be unbiased at probe-target divergence of up to 20% (Bonsall *et al.* 2015), providing a convenient approach for detecting and sequencing even highly diverse pathogens such hepatitis C virus (Bonsall *et al.* 2015, Ansari *et al.* 2019), and HIV (Bonsall *et al.* 2020, Jenkins *et al.* 2023), as well as for analysis of genetic variation in infection (Lythgoe *et al.* 2021) and for molecular profiling of cell-free tumour DNA (Alborelli *et al.* 2019).

A key feature of targeted enrichment is that the oligonucleotide panel can be customized to include organisms or genes of interest, forming an explicit set of hypotheses against which results can be assessed. This is in contrast with shotgun metagenomic sequencing, where the entire nucleic acid extract is sequenced, and expert judgement must be made about the potential clinical significance of every identified organism.

Although hypothesis-free analysis tools designed for shotgun metagenomics data, such as QIIME (Bolyen *et al.* 2019), MetaPhlan (Blanco-Míguez *et al.* 2023), and Kraken2 (Wood *et al.* 2019), can be repurposed for the analysis of targeted enrichment data, these are designed simply to quantify and report relative taxonomic abundance and/or richness in the sample, rather than to detect a finite set of organisms of interest.

From an analysis perspective, targeted sequence data have several attractive features that distinguish it from shotgun metagenomic data. First, the panel of targets is pre-defined, making it possible to use established, highly efficient reference-based mapping strategies for sequence processing, with only minor modifications. Second, since a background level of (un-targeted) metagenomic reads remains in the sequenceable library, the difference in read depth between the targeted and untargeted sequences can be used to calculate efficiency of capture, helping to distinguish true low-abundance positives from low-level background reads. Targeted sequencing combined with reference mapping also allows for read de-duplication, and therefore lends itself to quantitative analysis of input material, equivalent to estimating pathogen load directly from sequence data, as has been demonstrated for estimation of pathogen abundance in HIV (Bonsall *et al.* 2020), RSV (Lin *et al.* 2021, 2024), and other organisms (Goh *et al.* 2019). Although these features of targeted sequencing are likely appreciated within laboratories that use capture, wider uptake of targeted sequencing requires bioinformatics tools to support the specific features of the laboratory method.

Here, we present the Castanet pipeline (hereafter ‘Castanet’), an end-to-end, automated pipeline specifically designed for the analysis of targeted sequencing data. Our motivation was to create an efficient, easy-to-use, modifiable pipeline that enables rapid processing of a targeted sequencing experiment, and outputs all relevant metrics for downstream analysis. Castanet produces consensus sequences for targets identified in the data, along with summary statistics per target and per organism. It is driven by a robust workflow management system based on an application programming interface (API), which coordinates data versioning, output data management, testing, error management, and concurrency features.

User input to Castanet consists of paired read files (FASTQ with or without compression) and a mapping reference (FASTA file) containing one or more targets of interest. An option exists for users to supply pre-mapped reads (BAM format instead of FASTQ).

**Figure 1.**
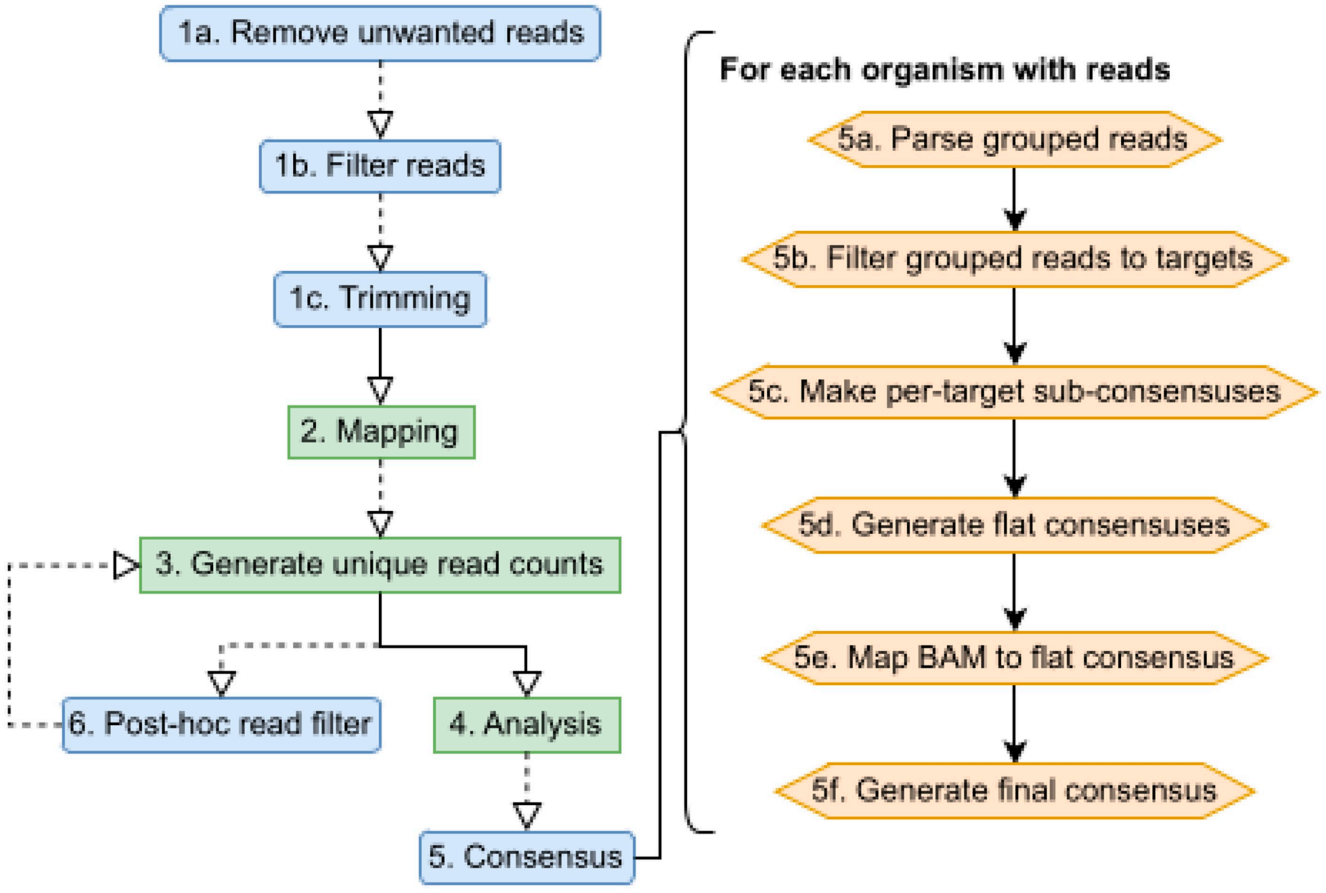
Castanet process flow. (Left) Castanet pipeline. Blue boxes are optional stages. (Right) Consensus generator algorithm.

Castanet is based on several open source tools, as well as additional methods for classification and quantification (Fig. 1) based on aggregation of reads by organism across multiple target sequences. This depends on an initial mapping to a collection of target sequences (the ‘mapping reference’). The reference will normally reflect the set of targets against which the capture probes were designed, although need not match precisely, as the selective amplification of specific targets enables prior assumption of sequence composition within a sample. Control strategies can be supported; for example, a reference sequence that contains both a targeted and an untargeted region can be used to estimate enrichment efficiency.

Castanet’s analytical functions are species-agnostic and examine the comparative distribution of duplicated and deduplicated (unique) reads and their genomic positions. This allows for estimation of pathogen abundance, analysis of capture efficiency and efficient elimination of background reads. Aggregation of reads mapped to multiple targets to the level of individual organisms is achieved by interpretation of target nomenclature via natural language processing functions, according to defined rules. Statistics are then generated for every organism where reads have been detected, including coverage, number of targets/loci mapped, amplification rate, and read proportions.

Consensus sequences are additionally generated where coverage is sufficient, via an algorithm that generates ‘sub-consensuses’ for each target before they are flattened (taking majority bases from a partial alignment of reads and reference sequence) and reads are re-mapped to this as a reference. This alignment is used to generate the final ‘remapped consensus’, using ViralConsensus (Moshiri, 2023). The workflow is amenable to generating bacterial, fungal and parasite consensus sequences, as well as viruses. Crucially, Castanet’s consensus reconstruction does not derive spans of consensus sequences from their references, which allows for more precise downstream analyses such as strain typing.

Castanet was originally written as an in-house research tool (Goh *et al.* 2019), implemented in Python 2 (https://github.com/tgolubch/castanet); this experimental version has been publicly available since 2019 and has been in extensive use within our own laboratories since 2018. To maximize the utility of the software for the bioinformatics community, we have now updated, optimized, fully automated and substantially expanded functionality to include consensus calling and utility functions. Aims for the Castanet application included:

- *User friendliness*. Extensive bioinformatics experience is not required to install and run the software. Users may run jobs from either a simple command-line interface (CLI) or a browser-based graphical user interface (GUI). An API is available for expert users. Documentation is provided as a Wiki in the GitHub repository.
- *Continuous Integration/Continuous Development (CI/CD) and Workflow management*. Castanet is containerized with Docker and automatic build scripts are included for local installations. The API workflow model enables reproducibility, robustness and scaling features and a combination of pre-commit Git hooks and PyTest suite ensures code quality.
- *Automation*. No manual curation is needed to run Castanet other than ensuring the mapping reference is generated as a standard multi-fasta file with an appropriate sequence naming convention.
- *Performance*. A paired read file of approx. $1\times 10^{6}$ reads may be analysed in under 5 minutes, on a consumer-grade laptop with a 16 thread 3.30 GHz processor and 32 Gb RAM.

Here we detail experiments validating Castanet and conclude by critically evaluating results in the context of the current gold standard methods.

## 2 Materials and methods

### 2.1 Software

Castanet is written in Python 3.10 and is native to Ubuntu 22.04, but is also compatible with all Linux-like environments including Mac. The application is hosted on a lightweight Uvicorn server behind which workflows are managed by a RESTful API composed in FastAPI (https://github.com/tiangolo/fastapi). The application also has a simple command-line interface (CLI). Users may opt to use the CLI, or to run jobs from the standalone dashboard via a browser. All external dependencies may be installed via a shell script included in the repository. A full README and documentation WIKI are included in the repository.

Individual pipeline stages (Fig. 1) are detailed below, with external dependencies listed in bold. The pipeline may be run as a single end-to-end process; a batch process that will iteratively fire end-to-end runs for all samples within a single directory; or individual pipeline stages. Users may, for example, skip steps 1–4 if they already have a BAM file of reads mapped against their chosen reference.

1. (Optional) Pre-process data:
  - Taxonomically classify reads (call **Kraken2**).
  - Filter out unwanted reads based on taxonomy, e.g. arising from the host genome.
  - Trim adapters and remove low quality reads (call **Trimmomatic**; Bolger *et al.* 2014).
2. Map cleaned reads to reference sequences to produce a BAM file containing all mapped reads, including improper pairs (call **BWA-mem2**; Vasimuddin *et al.* 2019, **SAMtools**).
3. Parse BAM file:
  - Aggregate reads by target. Remove spurious matches falling below a user-defined threshold on fragment length (TLEN). Improperly paired reads are allowed if the paired mate matches another variant of the same target (e.g. another variant of a targeted virus), and is of sufficient mapped length.
  - Generate total and unique (position-deduplicated) read counts.
  - Temporarily cast reads grouped by target to separate BAM files.
4. Call analysis functions:
  - Parse reference lengths, merge with position counts.
  - Aggregate target sequences as organism-locus, via regular expressions. Organism-locus can be understood as a group of non-homologous regions that comprise the entirety of targeted sequence for a specific organism, e.g. multiple genes from a bacterial genome.
  - Generate statistics for each organism-locus.
  - Generate approximate coverage plots for each organism-locus.
5. Reconstruct consensus sequences (Fig. 1). Gaps in genomes with incomplete coverage are not filled by the reference. For each organism with reads:
  - Parse all reads grouped by target from step 3.
  - For each organism with reads, filter reads to each individual target, via collation from step 4.
  - Generate per-target ‘sub-consensus’ sequences (**SAMtools**).
  - Create ‘flat consensus’: Filter master BAM to organism-specific targets with coverage and depth exceeding user-specified thresholds and align sub-consensuses with relevant reference sequences, then generate a consensus (**SAMtools, Mafft** (Katoh and Standley 2013)).
  - Remap filtered master BAM from step 2 to flat consensus (**BWA-mem2**). RGB LED Tape Extension Lead.
  - Call final consensus on remapped flat consensus (**ViralConsensus**).
6. (Optional) Output of analysis (misassigned reads) used to filter input BAM file, re-call step 3 (**SAMtools**).

### 2.2 Experimental evaluation

To evaluate Castanet, we used five datasets: Two synthetic and three real (Table 1). To focus the testing strategy on what is a primary use of Castanet in our laboratory, all experiments focused on detection of viral agents. We used a mixture of data with unknown (set A) and known (set B) genomic content to compare Castanet’s utility in both ‘real-world’ and benchmark contexts.

**Table 1.** Dataset descriptions, excluding viral multiplex control.^a^

Expected organism	Set	Genome length (bp)	*N* targets	*N* samples	Method	Specimen type
Viral multiplex control	A.1	Various	5	5	Target enrichment	Reference material
Negative plasma	A.2	N/A	N/A	5	Target enrichment	Plasma
Marburg virus	B.1.1	1.9 × 10^4^	5	3	Synthetic	N/A
HCV-1A, 2A, 2B	B.1.2	∼9.4 × 10^3^	3	3	Synthetic	N/A
RSV-A, B	B.2.1	∼1.5 × 10^4^	12	17	Target enrichment	Nasopharyngeal swab
HIV-1	B.2.2	∼9.7 × 10^3^	9	21	MTA-Seq	Whole blood

a N targets: number of target sequences to map against in probe set. MTA-seq: multiplex PCR targeted amplicon NGS sequencing.

For set A (ENA PRJEB77004), Castanet was used to analyse a dataset generated in-house (Supplementary Document 1, S1) via target capture as a simulated clinical application and included:

A.1 Viral multiplex reference (National Institute for Biological Standards and Control, London, United Kingdom). Pre-mixed material from 25 viruses of human and bovine origin, of which the following were quantified in International Units: Parechovirus (HPeV), human herpes virus 4 (HHV4, more commonly known as Epstein-Barr virus, EBV), human herpes virus 5 (HHV5, more commonly known as cytomegalovirus, CMV). This control was included as a dilution series to support estimation of viral load.
A.2 Five pooled clinical plasma samples from healthy donors, confirmed as PCR-negative for multiple infectious agents including HIV, Hepatitis B, C, and E. These were included as being representative of negative samples in a screening context that nevertheless could contain low numbers of reads for adventitious, non-pathogenic viruses such as torque teno virus (TTV).

Set B included both simulated (set B.1, Supplementary Document 1, S2) and publicly available data (set B.2). These included:

B.1.1 Marburg virus is a filamentous negative-sense ssRNA virus with three distinct clusters where intra-species nucleotide divergence is <20% (Scarpa *et al.* 2023). This represents a straightforward use case where retrieval of the entire genome is feasible through use of a small number of probes.
B.1.2 Hepatitis C virus (HCV), equal mixture of reads from types 1a, 2a, and 2b genotypes. This simulates a complex but relatively common clinical issue where multiple viral subtypes or quasi-species may co-exist within a patient (Smith *et al.* 2010). As a short positive-sense ssRNA virus, full genome coverage may easily be achieved with a small number of probes.
B.2.1 RSV. 17 clinical respiratory samples extracted from nasal swabs, in infants infected with this linear negative-sense ssRNA virus (ENA ERR10812874–86) (Lin *et al.* 2024). Samples were collected from UK and Spanish infants aged less than one year during the 2017–18 RSV season for the purpose of investigating virus subtype distribution and differential gene expression in this outbreak. The original study identified and published several diverse, novel isolates through a curated workflow using Shiver (Wymant *et al.* 2018); the current study did not omit the four samples that the original study’s authors were not able to extract reference sequences from.
B.2.2 HIV-1. 21 clinical blood samples from individuals known to be HIV-1 positive, collected as part of a pan-European project (ENA ERR732065–90) for the purpose of providing open source data for HIV researchers (Hunt *et al.* 2015, Wymant *et al.* 2018). HIV is retroviruses possessing duplicate copies of positive-sense ssRNA genomes whose capability for rapid mutation frustrates efforts to treat and study it (Hargrave *et al.* 2021); hence, reconstruction of HIV genomes from NGS data is challenging because an individual with HIV is likely to be host to multiple quasi-species of the virus with significant intra- and inter-host divergence. HIV-1 sequences were pre-determined using a simple pipeline of Iterative Virus Assembler (IVA) (Hunt *et al.* 2015) followed by Shiver, using default settings for both.

Descriptive statistics were collected including number of mapped reads, mean depth, coverage at depths 1 and 10, mean amplification rate (MAR, number of reads ÷ number of deduplicated reads at each position supported by reads), and consensus sequence quality were recorded from Castanet’s default output. Consensus quality was measured by comparing Castanet’s output with published reference sequences, using BLASTn (query coverage and percentage identity, hits where E values <0.01) and Mash scores (Ondov *et al.* 2016). The rationale for adopting two metrics was that Mash is a more sensitive tool and hence provides more information in instances where BLAST scores are very high.

We used an in-house mapping reference of 3682 sequences against a range of clinically relevant viral, fungal, parasitic, and bacterial targets, including ribosomal multi-locus (rMLST) (Jolley *et al.* 2012) targets for the latter, in all experiments. Castanet’s default settings were used throughout.

#### 2.2.1 Hardware

All experiments were conducted on a laptop with 16 hyperthreading-enabled cores (i9-11980HK, 3.30 GHz), 32 Gb DDR4 RAM and M.2 SSD with read/write speed of $6\times 10^{6}$ Mb s^−1^, running Windows Subsystems Linux Ubuntu 22.04 LTS. Experiment run time was benchmarked in all experiments.

## 3 Results

The average run time for a single sample across all experiments conducted between sample sets A–B was 7.28 min (mean read depth $5.2\times 10^{5}$), the longest run in 31.0 min which comprised over $3\times 10^{6}$ reads and involved generating consensus sequences for four organisms. Benchmarking is included in the SI (Supplementary Document 1, S3).

**Figure 2.**
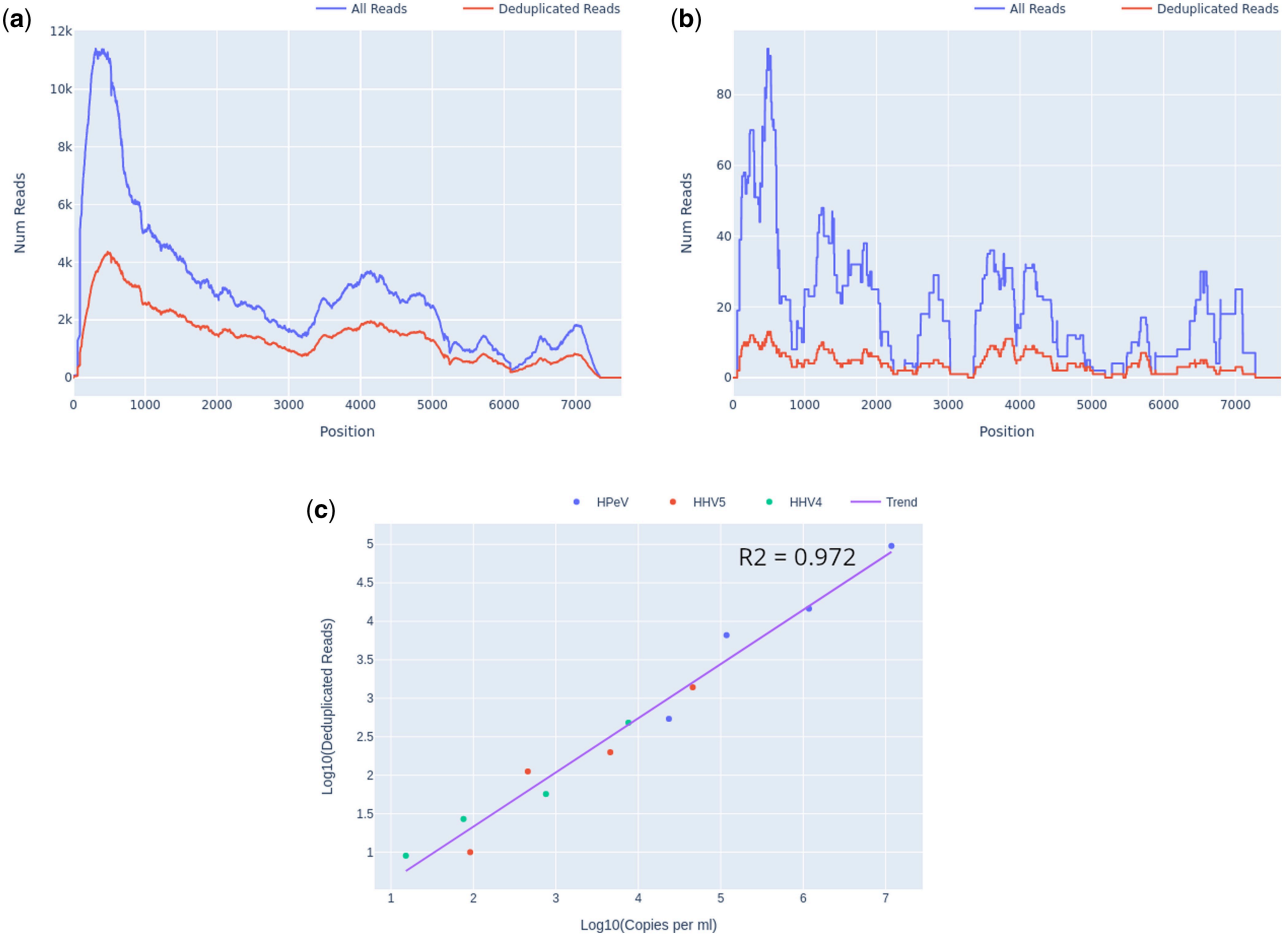
Castanet output for set A.1, at several dilutions. (a) Read depth graph showing total and deduplicated (unique) reads for HPeV, undiluted, viral load 9.6 × 10^4^ copies per ml. (b) Read depth, HPeV, 1:500 dilution, 5.3 × 10^2^ copies per ml. (c) Log10 deduplicated reads versus Log10 copies per ml for HPeV, HHV4, and HHV5 at four different dilutions (1, 1:10, 1:100, and 1:500), with linear regression line.

For detected organisms of interest, plots of read depth across the span of each genome for both deduplicated (unique) and total reads were generated automatically by Castanet (Fig. 2a and b). These were used to assess coverage and, in the case of pre-quantified controls, estimate viral loads (Fig. 2c) using linear least squares regression of log_10_ of deduplicated reads reported by Castanet versus log_10_ of known laboratory-reported viral load from qPCR experiments, in the three viruses from set A.1 (HPeV, EBV, and CMV).

**Figure 3.**
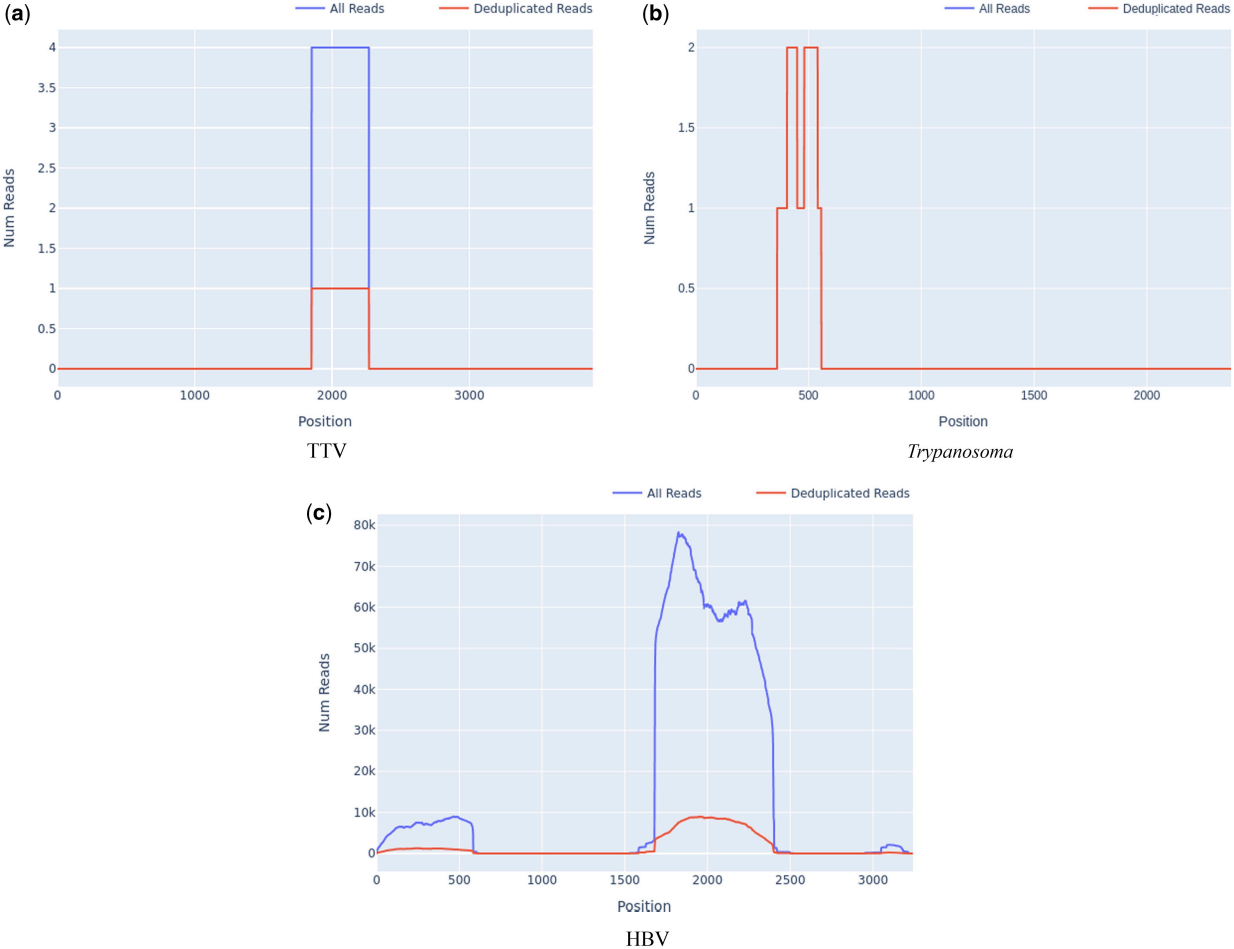
Appearance of presumptive low positives, false positives, and contamination in read depth plots from set A.2. (a) Reads for TTV, with high amplification rate across the region with reads (4.0), across a partial region of the genome. (b) *Trypanosoma* spp. reads at the ribosomal 18s locus, with no amplification and incomplete coverage. (c) HBV reads with incomplete coverage and significant amplification (MAR 5.82, SD 3.37).

A significant benefit of targeted sequencing is that signal of enrichment may be useful for distinguishing true low abundance positives from the background sequences that are a ubiquitous feature of all sequencing experiments (Jurasz *et al.* 2021). In our experience, genome coverage plots of low-abundance true positives that have been successfully sequenced with target enrichment are expected to show a characteristic “city-block” appearance, with a series of highly amplified fragments across the targeted regions, which result from capture and amplification of a small number of template fragments in the sample. We observed such patterns in both controls at known low titres (Fig. 2b) and clinical samples where low pathogen abundances were anticipated, e.g. commensal viruses such as TTV (Fig. 3a). In contrast, background sequence noise, such as index misassignment or carryover from other samples in the batch, will have very few or no duplicated reads at the targeted region, with no evidence of amplification which is what we observed (Fig. 3b). It is notable that the use of amplification intensity to distinguish low-abundance positives from background noise is method-specific; it is well suited to targeted enrichment protocols and generally does not apply to shotgun metagenomics, allowing for certain edge cases, e.g. index misassignment, contamination from another library. Consequently, when untargeted shotgun data are analysed with Castanet, the plots can be expected to show very little sequence duplication (Supplementary Document 1, S4).

A common source of difficulties with standard taxonomic classification tools for metagenomics data, which quantify taxa on the basis of read identity, is that reads may originate from a high-abundance contaminating source, such as pre-amplified PCR product. In standard metagenomics processing, such contaminated samples will yield extremely high numbers of reads matching a given taxon and will be considered high-confidence positives. However, using reference mapping, contamination of the sample, e.g. pre-amplified (PCR) product is readily detectable as unusually high depth across a defined proportion of the targeted region, with no evidence of sequence across the rest of the targets for the same organism. An example of this type of contamination is shown in Fig. 3c, where an HBV sample was accidentally contaminated by a small amount of amplified product from a diagnostic PCR in the originating laboratory, seen as a discrete region of high depth at the targeted genomic region, and a smaller region of cross-mapping of some reads to an imperfect genomic repeat of the target. Castanet uses mapping to make this analysis intuitive.

**Figure 4.**
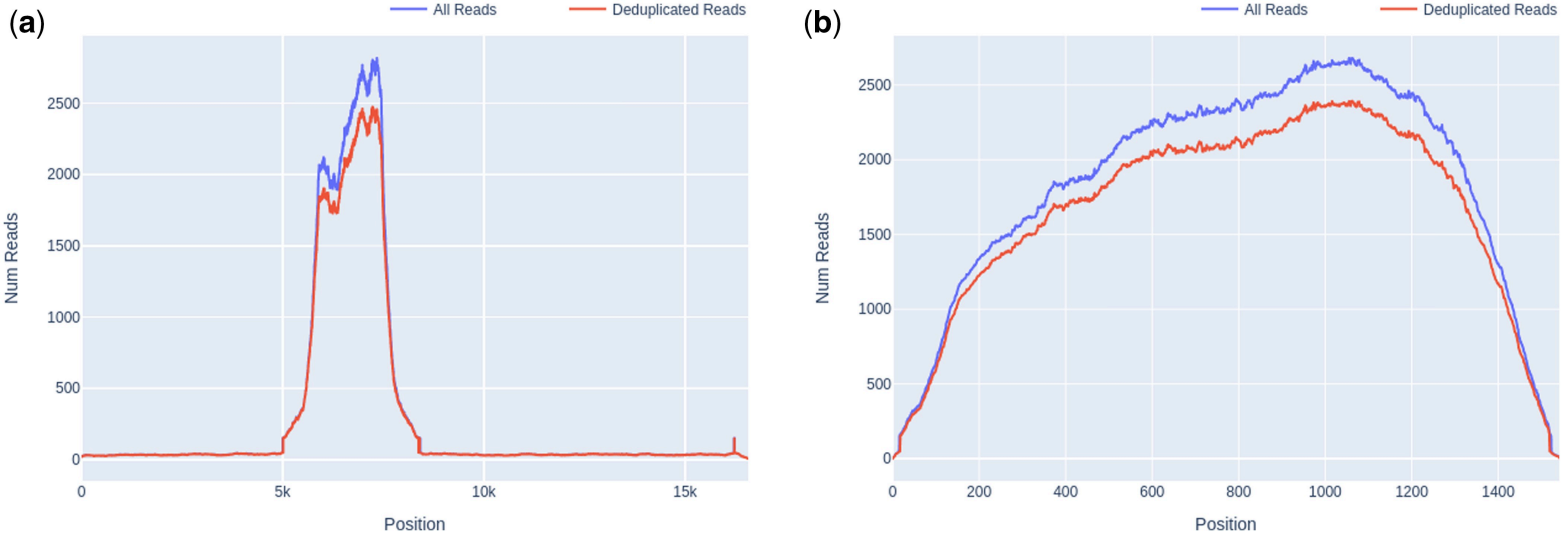
Comparison of read depth between untargeted and targeted sequences, in a screened pooled human plasma sample (set A.2). (a) Whole human mitochondrial gene, median depth 16 (excluding COX-1 region). (b) Targeted sequence, mitochondrial COX-1, median depth 2109.

Another crucial process in analysing a target enrichment experiment is estimating the efficiency of capture, as many downstream applications rely on amplification of target sequences being sufficient to achieve adequate coverage and read depth to properly represent sample diversity. This may be achieved with Castanet’s output in several ways, including by comparing the mean sequencing depth between targeted and non-targeted sequences. Non-targeted sequences are part of the uncaptured (metagenomic) background, and hence would be expected to have little-to-no evidence of read duplication, whereas targeted sequences should have significant amplification (100–1000× the background) in cases where capture has been successful. In a representative example from set A.2, we found the read depth to be ∼130× that of the metagenomic background when comparing read depth of the whole human mitochondrial genome (Fig. 4a) with cytochrome C oxidase I (COX-1) (Fig. 4b), where the latter was the only targeted region in the former.

A primary novelty of Castanet is its aggregation of reads to the correct organism, which it did correctly in all experiments from set B (Table 2). For consensus reconstruction, counts as low as $1\times 10^{4}$ were sufficient to recover the vast majority of genomic content in all experiments. For detection of organisms where a complete genome is not required, a significantly lower read number (approximately $1\times 10^{2}$) was sufficient.

**Table 2.** Summary statistics for Castanet validation on set B data.^a^

	Marburg virus			HCV-1A/2A/2B				
	1 × 10^4^	$1\times 10^{5}$	1 × 10^6^	3 × 10^4^	$3\times 10^{5}$	$3\times 10^{6}$	HIV-1	RSV-A, B
*n*	10	10	10	10	10	10	21	17
$\bar{x}$ run time	0.5	1.0	10	1.2	3.1	29	6.5 (4.7)	8.5 (9.0)
Con Rec (%)	100	100	100	100	100	100	100	76
$\bar{x}$ N reads	0.9 × 10^4^	1.0 × 10^5^	1.0 × 10^6^	3.0 × 10^4^	3.0 × 10^5^	3.0 × 10^6^	8.2 × 10^4^ (1.1 × 10^4^)	7.0 × 10^4^ (3.1 × 10^4^)
$\bar{x}$ depth	2.6 × 10^2^	2.6 × 10^3^	2.6 × 10^4^	1.5 × 10^3^	1.5 × 10^4^	1.5 × 10^6^	2.6 × 10^4^ (1.7 × 10^4^)	1.4 × 10^4^ (1.9 × 10^4^)
$\bar{x}$ Amp rate	1.00	1.02	1.16	1.00	1.03	1.33	3.35 (1.39)	2.98 (1.93)
$\bar{x}$ Cov 1 (%)	99.9	99.9	99.9	98.1	98.2	98.2	98.32 (0.55)	96.1 (6.71)
$\bar{x}$ Cov 10 (%)	99.8	99.9	99.9	98.1	98.2	98.2	94.83 (7.26)	83.7 (26.6)
$\bar{x}$ Con QC (%)	90.0	99.0	99.1	99.0	100	100	92.3 (2.86)	98.3 (1.55)
$\bar{x}$ Con PcID (%)	99.9	99.9	99.9	99.9	100	100	98.6 (1.07)	100 (0.02)
$\bar{x}$ Con MH (%)	84.2	95.4	95.6	99.7	100	100	63.2 (16.1)	98.7 (1.89)

a $\bar{x}$ : mean; parentheses indicate standard deviation on set B.2 (real) datasets. Con Rec: percentage of samples where consensus was recovered; N reads: number of reads on target; Depth: read depth; Amp rate: amplification rate (*n* reads ÷ *n* reads deduplicated); Cov: percentage coverage with depth >1/>10; Con QC: consensus BLASTn query coverage versus reference sequence; Con PcID: consensus BLASTn percentage identity versus reference sequence; Con MH: consensus Mash score versus reference sequence.

The quality of consensus sequences, measured via BLASTn and Mash scores comparing Castanet consensuses to reference sequences, increased to peak values exceeding 99% (BLASTn QC/PcID) and 95% (Mash) at read counts exceeding $1\times 10^{5}$ in set B.1. We were able to differentiate between and generate consensus sequences that approached 100% identity with reference sequences, for all three HCV subtypes in set B.1.2. Reads on target (i.e. mapped to the reference) in the order of $10^{4}$ were retrieved in both B.2 datasets, which was sufficient to generate coverage exceeding 83% in all cases, or 94% if excluding the four insufficient RSV samples. These four RSV samples had much lower mean coverage (mean 85.5%, SD 6.20% and mean 45.0%, SD 25.4% at depth 1 and 10, respectively) and MAR than other samples, although consensus sequences with sufficient coverage to support strain typing were generated for all, where this had not been achieved previously.

## 4 Discussion

Castanet is designed to provide outputs of greatest interest to those running targeted multi-organism sequencing experiments, but is broadly applicable to sequence data generated using other methods. Reporting of statistics such as coverage and depth is essential in any sequencing workflow, as these indicate the relative success of the experiment and whether downstream analyses such as variant discovery will be possible. Castanet reports additional statistics such as MAR (an amplification of 1 indicates poor enrichment, or no enrichment or contamination): we used this in set B.2.1 experiments to demonstrate correlation of failure to recover a consensus genome with low amplification, which could result from experimental factors such as sample insufficiency. The full Castanet analysis output (Supplementary Spreadsheet 1, Sheet 1) encompasses 51 statistics which allow for flexible characterization of experimental data depending on experimental design.

Although we focused our reporting on the most abundant organisms in the samples described here, a range of other organisms were detected in the majority of B.2.1 samples including expected viral and bacterial species (human rhinovirus, *Moraxella catarrhalis*, *Haemophilus influenzae* among others) (Supplementary Spreadsheet 1, Sheet 2). This highlights the utility of Castanet for rapid and comprehensive analysis of target capture data to characterize a wide variety of organisms in a single workflow which could be applied to diagnostic microbiology settings.

The quantitative relationship between deduplicated read counts and a sample’s viral load, described extensively in previous publications (Bonsall *et al.* 2020, Lythgoe *et al.* 2021, Lin *et al.* 2024), further illustrates how Castanet’s output may feasibly be used in conjunction with target capture methods to complement or, in specific circumstances, replace PCR testing in a diagnostic setting, provided sufficient quality control and calibration provided for as part of the workflow. While it is beyond the scope of this study to estimate the time and monetary cost of such a move, contemporary studies have demonstrated that NGS may offer significantly reduced costs over PCR when deployed at scale when analysis of multiple targets is required, such as cancer biomarker testing (Bestvina *et al.* 2024), mass HIV screening (Bonsall *et al.* 2020), and *Mycobacterium tuberculosis* testing in public health settings (Mann *et al.* 2023).

Contamination is a recognized risk in all high-throughput sequencing workflows, with multiple potential sources for it to be introduced into a library. Deriving the consensus sequence directly from initial analysis can help troubleshoot the likely source of contamination, e.g. in the case we examined (Fig. 3a), the contaminant was traced to PCR amplicon from a neighbouring laboratory source. We have found that a coverage proportion at minimum depth 2 of ∼10—15% to be a good indicator of likely true presence in problematic samples with low read depth, and >30% is usually confirmatory, although these precise thresholds will differ between laboratories, methods, and target organisms, so should not be considered without reference to the amplification rate. This highlights another justification for the use of target capture methodologies in high-throughput settings when leveraged via Castanet.

Users may evaluate coverage per-target and fine-tune consensus generation parameters where necessary, using the automatically generated consensus alignment plots (Supplementary Document 1, S5). This is relevant in cases such as set B.1.2, as it was known *a priori* that three distinct HCV variants were present. Default Castanet behaviour would aggregate all reads to “Hepatitis C” and generate a consensus sequence represented by the majority subtype. There are multiple options for extracting more precise genotyping data from Castanet, such as modification of mapping reference nomenclature, use of a separate mapping reference file where aggregation is allowed only at the level of subtypes, or extracting multiple sub-consensuses from the consensus module’s output. It may be sufficient for most workflows, however, to confirm the presence of reads against a specific organism with Castanet and use the consensus sequence to compare bulk genomic data between samples before doing downstream strain typing.

All consensus quality scores were lower in set B.2.2 experiments (against set B.2.1, consensus query coverage 92.3% versus 98.3%, percent identity 98.6% versus 100%, Mash score 63.2% versus 98.7%), as would be expected for viruses such as HIV-1 that form diverse intra-host populations, as alignment involves taking majority values across all sub-consensuses. In such cases, it is possible to extend Castanet to add a species-specific workflow once the correct pathogen has been identified. HIV-1 data used for benchmarking in this study were generated with MTA-seq, which highlights the utility of the Castanet pipeline for different NGS techniques.

Comparisons of Castanet with the software used to generate reference sequences are included here to guide users towards the appropriate choice of analytical pipeline. In the case of our most difficult example, HIV, shiver was specifically designed for optimal reconstruction of HIV genomes from highly diverse clinical samples, by regenerating missing regions from the closest-matching reference sequence from a user-defined library, or from *de novo*-assembled contigs. This requires a curated database of reference sequences and possibly expert input to evaluate the quality of alignments before contigs are flattened with the references. Castanet output is suitable for further processing with shiver or other software, but we chose not to implement additional specific workflows within the tool itself.

We have presented Castanet’s method of aggregating reads to specific targets at the level of organisms as a feature, but it is pertinent to note two limitations. First, the naming conventions of the user’s probe file must be kept consistent to ensure organism-level aggregation; three regular expressions are provided in Castanet’s documentation to guide users, and our future work includes further refinement of this step. Second, for consensus reconstruction, we assume genome collinearity with references, sample colonization by a meaningful majority quasi-species per organism, and a theoretical coverage of 100% across the targeted regions per organism. In cases where only part of an organism’s genome is targeted, we recommend that the user defines the reference sequences appropriately to match the targeted regions, or sets the consensus parameters in line with a reduced requirement for genome coverage. This is true particularly where larger genomes such as those of bacteria or fungi are only partially represented in a user’s target enrichment panel, and thus Castanet would not be expected to recover a complete consensus genome but only the sequences of targeted regions. In general, the reference should be provided in accordance with the design of the experiment. Multipartite genomes (e.g. segmented viruses or multiple arbitrary genes from a single species) may be processed as separate segments or concatenated into a linear approximation. We have automated multi-gene concatenation specifically for bacterial ribosomal MLST (rMLST) genes (Jolley *et al.* 2012). Future work includes expanded support for multi-locus targets and segmented viral genomes.

Targeted sequencing is well-suited to diverse applications in both research and clinical practice, including pathogen genomics and transcriptomics. The capacity of large diagnostic laboratories to perform sequencing has increased greatly over recent years. As costs continue to fall, targeted sequencing is likely to supersede methods such as 16S sequencing to become part of the routine diagnostic armoury in clinical settings as combined diagnostic and surveillance testing. The Castanet pipeline facilitates bioinformatic analysis to take place outside research facilities, as it addresses the lack of open-source, end-to-end tools designed specifically to exploit targeted sequencing data and hence supports wider adoption of these technologies. Our analysis here has demonstrated how the software may be used to support a wide variety of applications as well as evaluate run performance and troubleshoot laboratory issues, in a scalable and reproducible manner.

## Supplementary Material

btae591_Supplementary_Data
